# Mathematical simulation and prediction of tumor volume using RBF artificial neural network at different circumstances in the tumor microenvironment

**DOI:** 10.1177/09544119211028380

**Published:** 2021-07-10

**Authors:** Mehran Akbarpour Ghazani, Mohsen Saghafian, Peyman Jalali, Madjid Soltani

**Affiliations:** 1 Department of Mechanical Engineering, Isfahan University of Technology, Isfahan, Iran; 2 Department of Mechanical Engineering, K. N. Toosi University of Technology, Tehran, Iran; 3 Faculty of Mechanical Engineering, University of Tabriz, Tabriz, Iran; 4 Department of Electrical and Computer Engineering, University of Waterloo, Waterloo, ON, Canada; 5 Centre for Biotechnology and Bioengineering (CBB), University of Waterloo, Waterloo, ON, Canada; 6 Advanced Bioengineering Initiative Center, Computational Medicine Center, K. N. Toosi University of Technology, Tehran, Iran

**Keywords:** Mathematical oncology, angiogenesis, vascular tumor, hybrid tumor modeling, artificial neural network, radial basis function

## Abstract

Uncontrolled proliferation of cells in a tissue caused by genetic mutations inside a cell is referred to as a tumor. A tumor which grows rapidly encounters a barrier when it grows to a certain size in presence of preexisting vasculature. This is the time when it has to find a way to go on the growth. The tumor starts to secrete tumor angiogenic factors (TAFs) and stimulate preexisting vessels to grow new sprouts. These new sprouts will find their way to the tumor in the extracellular matrix (ECM) by the gradient of TAF. As these new capillaries anastomose and reach tumor, fresh oxygen is available for the tumor and it will reinitiate the growth. Number of initial sprouts, distance of initial tumor cells from the vessel(s) and initial density of the tumor at the time of sprout formation are questions which are to be investigated. In the present study, the aim is to find the response of tumor cells and vessels to the reciprocal effects of each other in different circumstances in the tissue. Together with a mathematical formulation, a radial basis function (RBF) neural network is established to predict the number of tumor cells at different circumstances including size and distance of initial tumors from the parent vessel. A final formulation is given for the final number of tumor cells as a function of initial tumor size and distance between a parent vessel and a tumor. Results of this simulation demonstrate that, increasing the distance between a tumor and a parent vessel decreases the number of final tumor cells. Specially, this decrement becomes faster beyond a certain distance. Moreover, initial tumors in bigger domains must become much bigger before inducing angiogenesis which makes it harder for them to survive.

## Introduction

Cancer is known as the second leading cause of death worldwide.^1^ Therefore, understanding the mechanisms of this uncontrollable disease is of vital importance. Rapid proliferation of these abnormal cells depends on different spatial and temporal scales^2^ that is affected by the tumor microenvironment and the structure of the surrounding tissue.^3^ As known, tumor growth includes three distinct levels.^2^ At first, a tumor grows while it is fed by existing vasculature in the host tissue. Since tumor cells proliferate rapidly and consume more oxygen, the surrounding vasculature is not able to meet nutritious requirements of the starving tumor cells. In the second stage, the tumor seeks a way to overcome oxygen deficiency by inducing vessel growth toward the tumor. The process of stimulating and growing new capillaries from pre-existing vessels is known as angiogenesis. The third and most fatal part of the growth is when the tumor has acquired its own vascular network. Unstable and leaky capillaries are not only a source of oxygen, but also a shortcut for tumor cells to breach into and be transported to the other parts of the body which is called metastasis. Understanding these distinct levels and dependence of tumor on surrounding tissue is crucial in defining the outcomes of a chemotherapeutic treatment.

For many years, scientists were struggling to find the underlying mechanisms of this devastating disease.^4,5^ Folkman was the first scientist who distinguished the different steps of tumor growth.^6^ He then proposed the hypothesis that tumors are not able to grow beyond a certain size in the absence of angiogenesis.^7^ Since then, many scientists and clinicians have been working on angiogenesis and vascularized tumors to fully capture the underlying phenomena and hinder tumor growth via obstructing the ways for tumor to provoke angiogenesis. A momentous part which is still obscure for scientists is the effects of surrounding tissue and existing vasculature. In vivo models are difficult and costly to implement. Moreover, all physical phenomena happening in the tumor microenvironment cannot be investigated by in vivo models. On the other hand, in vitro models make clinicians able maneuver on some precise and controllable parameters. For instance, it is possible to culture endothelial cells and to see if they respond to some stimulating factors or not. However, these models have drawbacks which make them limited in a variety of ways. Restrictions related to temporal and spatial scales inhibit utilization of experimental methods for tumor studies.^8^ The only choice remaining is using in silico models which make researchers able to test their hypotheses without considerable effort in comparison to experimental methods. In addition, when new findings are acquired in experiments, scientists refer to in silico models to clarify and illuminate the underlying stimulators for the phenomena. Therefore, scientists and researchers have recently been attracted toward mathematical models and it is believed that integrated methods are the most reliable and efficient ways to study tumor growth.^9^ Wide variety of methods, parameters, physical situations, and time scales could be modeled through in silico models.

According to Araujo and McElwain,^9^ Hill^10^ was pioneer in studying the tumor microenvironment mathematically. Following him, many scientists have studied avascular and vascular tumor growth using different approaches leading to important outcomes which have assisted clinicians in the battle against their nemesis. All in all, mathematical models are categorized into three main groups of continuum, discrete, and hybrid. Each method is accompanied by its pros and cons. Continuum methods are governed by Partial Differential Equations (PDEs) and treat tumor as ensembles of cells acting and moving together. Hence, this modality is able to capture macroscale interactions between tumor cells. On the other side of mathematical models lies discrete ones. These methods as known by their names, handle each single cell inside the tumor microenvironment. As a result, it is feasible to track each cell and decide about its destiny in the model. Nonetheless, in this case, some macroscale phenomena cannot be simulated. Moreover, as the number of cells increases, time, and cost of simulation increases concurrently. To overcome these difficulties, researchers have combined two methods and hybrid formulation is achieved. In this formulation, some part of the simulation is governed by PDEs and the other by discrete methods. In some cases, it is suitable to use continuum methods to capture the phenomena in macroscale which is costly and difficult by discrete models.

Different macroscale aspects of tumor growth have been investigated by continuum methods.^1,11–15^ Mechanical interactions, cell-cell adhesion, cell-matrix interactions, and ECM stiffness are studied by the so called method which occur in macroscales. On the other hand, most models use discrete framework to treat tumor growth numerically.^2,16–19^ These methods have reported intracellular and intercellular phenomena such as cell birth and death, cell deformation and cell-cell signaling. And also hybrid models which are a combination of both models is being used to study tumor. Scientific literature is rich of papers about different mechanical and chemical interactions among cells and the tissue. Dyson et al.^20^ studied the mechanical properties of ECM and cell-ECM interactions and fiber anisotropy on the morphology of tumor cells. Authors stated that different patterns for cell aggregates and collagen and medium were observed under different mechanical conditions. The results showed that fibers realign by cell migration. In addition, circular, elliptic, and stripe configurations for tumor cell aggregates were observed. Cho and Levy^11^ evaluated the treatment efficacy of cytotoxic and cytostatic drugs. They concluded that tumor cells were totally eliminated when both drugs were infused in the tissue simultaneously and relapse is prevented. Salavati et al.^21^ worked on a new formulation of vascular tumors and validated the results by experiments on mice. The model confirmed the necessity of angiogenesis in solid tumors and the model can be used as a basis formulation to study therapeutics in solid tumors. Santagiuliana et al.^22^ assessed the biomechanical effects of the tumor microenvironment on tumor growth over time. According to the authors, decreasing ECM stiffness and cell-ECM adhesion and increasing porosity of ECM increases tumor mass. Sefidgar et al.^23^ studied effects of ECM stiffness on Endothelial Cell (EC) migration speed and reported that the ECs migrate faster in intermediate ECM stiffness. Salavati and Soltani^24^ investigated impacts of proliferation rate on the overall rate of ECs movement using a Cellular Potts Model (CPM). They concluded that using variable proliferation rate for ECs, more realistic results could be achieved which matches well with experimental observations. Effects of tumor shape and size on drug delivery is studied by Soltani and Chen.^25^ The results where indicative of importance of tumor size in filtration flux (flow rate out of the vasculature per unit volume) and pressure inside tumor. Bazmara et al.^26^ studied EC proliferation and migration focusing on the effects of blood flow on EC phenotype alteration. The results showed that in the absence of blood flow and shear stress, a loop cannot maintain its stability and collapses at the end. Following this research, Bazmara et al.^27^ published a paper studying blocking of intracellular signaling pathways to inhibit lumen formation and EC proliferation and hence to prevent angiogenesis during tumor growth.

Smeared finite element method which is introduced by Kojic et al.^28,29^ and further studied by Milosevic et al.^30^ can be used to model mass transport between capillaries and tissue in the tumor microenvironment. Kremheller et al.^31^ used smeared representation of neovasculature to capture species transport in tumor-induced vasculature which is highly tortuous and contains tiny vessels. They compared the results obtained by their model to preexisting models to validate their results.

Computational studies have been beneficial to assess the efficiency of chemotherapy for cancer treatment. Ribba et al.^32^ used an age-structured cell cycle model to examine efficacy of anti-metastatic agents, called inhibitors of matrix metalloproteinases (MMPi). It was revealed that MMPi is not an effective treatment in advanced cancers. Billy et al.^33^ coupled a continuum model of angiogenesis with a continuum model of tumor growth to develop an age-structured cell cycle model and examine influence of endostatin overproduction on hindering angiogenesis. It was concluded that endostatin overproduction may suppress angiogenesis and tumor growth. Ribba et al.^34^ investigated the effect of chemotherapy on tumor growth using an age-structured model and demonstrated that physio-pathological parameters have higher impact on treatment efficacy than drug-related parameters. Lignet et al.^35^ studied the interaction of antiangiogenic drugs and chemotherapeutic agents and presented an optimal therapeutic strategy for combined usage of these two treatments.

Pamuk et al.^36^ proposed a mathematical model to study tumor growth. They studied onset of vascularization of tumors inside the tissue. Their results were in good agreement with experiments. Perfahl et al.^37^ studied effects of domain size and boundary conditions on tumor response. Their results proved that distance of tumor and parent vessel, initial structure of the vascular network are important in determining the final architecture of the vascular tumor. Grogan et al.^38^ performed a mathematical model of mouse cornea to study effects of cornea geometry on vascular network formation. They studied effects of geometry on VEGF concentration and vascular density in the domain. The results proved that selection of pellet location and geometry of the domain greatly influences angiogenesis. Modeling of avascular tumor growth, blood flow and vessel regression are absent in their modeling. Recently, Nikmaneshi et al.^39^ proposed an agent-based mathematical model to study avascular and vascular tumors. The authors surveyed abnormal blood conditions encountered in cancer patients. It is concluded that hyperglycemia, hyperoxemia, and hypercarbia can aid in tumor growth. Moreover, hypertension can also lead to malignant tumors.

One important feature of tumor growth which is a debate among scientists in spite of great advancements in in silico models is the behavior of tumor cells in different distances of parent vessels. Which environmental conditions can make cells more devastating and when clinicians can better suppress tumor growth during the growth? Gimbrone et al.^40^ experimentally studied tumor growth in different conditions of parent vessel. Their results were helpful in understanding the states and reaction of tumor cells in the tissue. Recently, Ghazani et al.^41^ studied growth of a solid tumor at different distances of a parent vessel using a discrete tumor model where effects of tumor interstitial pressure in tumor cell cycle are also included.

In this paper, the aim is to make a useful contribution in understanding the fatality of a tumor in a variety of situations using a continuum tumor model and artificial neural network.

Effects of tumor location in the domain, domain size, and number of initial sprouts on the final architecture of the tumor and capillary network using a continuum tumor model and an agent based probabilistic angiogenesis method are studied.

## Mathematical model

In order to model growth of a tumor in a tissue, a hybrid continuum-discrete model of tumor growth is prepared. The mathematical model for simulation of tumor cells is a continuum age structured model which makes use of PDEs. A discrete agent-based method which comprises probabilistic phenomena is used to make us able to track each single EC in the domain. Therefore, branching, anastomosis and blood flow are considered in the model. Based on blood flow, oxygen diffuses in the domain and reaches nearby tumor cells. On the other hand, VEGF as dominant TAF is secreted by tumor cells and consumed and used by ECs to route their way.

### Tumor growth

**Figure 1.**
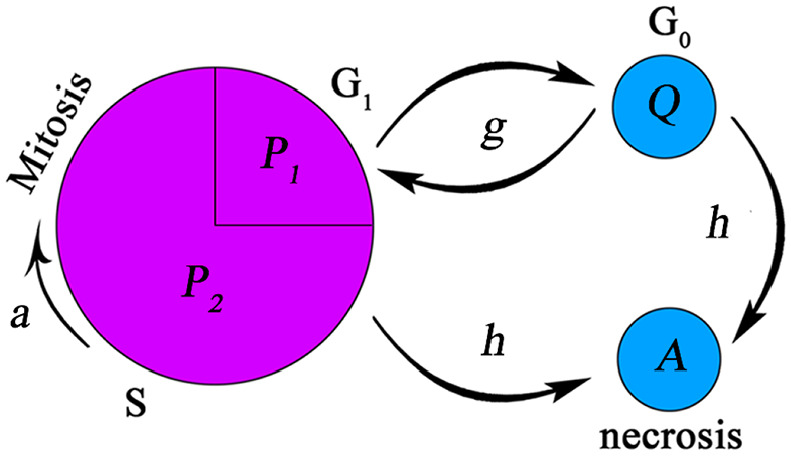
Schematic presentation of different steps in cell cycle. The restriction point is modeled at the end of *P*_1_ phase where environmental conditions are checked. Cell duplication occurs at the end of *P*_2_ phase. The function *g* determines phase transitions between proliferative and quiescent phases. The arrows show that cells can return to proliferative phase from quiescent phase. The function *h* is an indication of necrosis and the single arrow shows that cells entering the necrotic phase cannot exit the phase.

Tumor growth is modeled by a continuum age-structured cell cycle model embedded in a macroscopic model of tumor dynamics.^33^ Four different cell types are in tumor microenvironment: Proliferative, quiescent, necrotic, and healthy cells. To model mitosis, proliferative cells are considered to be in two different phases named proliferative phase 1 and proliferative phase 2. In “Restriction Point,” oxygen concentration and available spaces are evaluated to define where tumor cells are placed in their cell cycle. Cells enter proliferative phase 2 when oxygen concentration is sufficient for proliferation and space is available for cells to duplicate. As soon as cells pass the restriction point and enter proliferative phase 2, they continue their cell cycle disregarding oxygen availability. If defined conditions are not satisfied, cells are transferred to quiescent state. Cells can survive unlimited in the quiescent state until the conditions are met. If oxygen concentration falls below severe hypoxia threshold, the cells cannot endure severe oxygen deficiency and become necrotic (the condition is modeled as function *h*). Therefore, proliferative cells develop in both age and time in their cell cycle while quiescent and necrotic cells develop only in time. Figure 1 shows schematic of cell cycle.

To model transition between different phases in tumor microenvironment, functions *g* and *h* are introduced as follows in point (*x*, *y*) and time *t* in equations (1) and (2).

(1) $$g(x,y,t)=\left\{ \begin{array}{cc} 1 & \mathrm{if}\;\int_{0}^{a_{\max,P_{1}}}P_{1}\left(x,y,t,a\right)\;da+2\;\\ & \;\int_{0}^{a_{\max,P_{2}}}P_{2}\left(x,y,t,a\right)\;da+Q\left(x,y,t\right)\leq \tau_{0}\\ & \;\mathrm{and}\;O\left(x,y,t\right)\geq \tau_{1,h}\\ 0 & \mathrm{else} \end{array}\right.$$

(2) $$h\left(x,y,t\right)=\left\{ \begin{array}{cc} 1 & \mathrm{if}\;O\left(x,y,t\right)\geq \tau_{2,h}\\ 0 & \mathrm{else} \end{array}\right.$$

where *P*_1_, *P*_2_, *Q* are proliferative phase 1, proliferative phase 2 and quiescent cell’s density, *O* is oxygen concentration, τ_*O*_, τ_1,*h*,_ and *τ_2,h_* are overpopulation threshold, hypoxic threshold and severe hypoxic threshold, *a* is the cell’s age in time, and $a_{\max,P_{1}}$ , $a_{\max,P_{2}}$ are maximum duration of proliferative phases *P*_1_ and *P*_2_, respectively. Since there are two proliferative phases in the model, total density of proliferative phase is calculated in point (*x*, *y*) and time *t* in equation (3).

(3) $$\begin{array}{c} P(x,y,t)=\int_{0}^{a_{\max,P_{1}}}P_{1}\left(x,y,a,t\right)\;da+\int_{0}^{a_{\max,P_{2}}}\\ \;P_{2}\left(x,y,a,t\right)\;da \end{array}$$

where *P* is the total density of cells in proliferative phase. Since the total number of cells per unit volume is constant, densities are non-dimensionalized by constant total number of cells, *N_0_*, as shown in equation (4).

(4) $$\begin{array}{l} P\;+\;Q\;+\;A\;+\;M\;=\;N_{0}\;\Rightarrow \;P/N_{0}\;+\;Q/N_{0}\;+\;A/N_{0}\;+M/N_{0}\;=\;1\\ \tilde{P}+\tilde{Q}+\tilde{A}\;+\;\tilde{M}=1 \end{array}$$

where *A* and *M* are densities of necrotic and healthy phases, respectively. Tilde, (∼), is omitted in equations (5) and (6) for the sake of brevity. Tumor cells develop in both age and time in proliferative phases, therefore the evolution equations are presented in equations (5) and (6).

(5) $$\frac{\partial P_{1}}{\partial t}+\frac{\partial P_{1}}{\partial a}+\nabla .\left(v_{P_{1}}P_{1}\right)=0$$

(6) $$\frac{\partial P_{2}}{\partial t}+\frac{\partial P_{2}}{\partial a}+\nabla .\left(v_{P_{2}}P_{2}\right)=0$$

Quiescent and necrotic cells develop in time only in equations (7) and (8).

(7) $$\begin{array}{c} \frac{\partial Q}{\partial t}+\nabla .\left(v_{Q}Q\right)=h\left(1-g\right)P_{1}\left(a=a_{\max,P_{1}}\right)\\ \;-{\left({g\prime }_{t}\right)}^{+}Q\left(t^{-}\right)+{\left({h\prime }_{t}\right)}^{-}Q\left(t^{-}\right) \end{array}$$

(8) $$\frac{\partial A}{\partial t}+\nabla .\left(v_{A}A\right)=\left(1-h\right)P_{1}\left(a=a_{\max,P_{1}}\right)-{\left({h\prime }_{t}\right)}^{-}Q\left(t^{-}\right)$$

where ${\left({g\prime }_{t}\right)}^{+},{\left({h\prime }_{t}\right)}^{+}$ and ${\left({g\prime }_{t}\right)}^{-},{\left({h\prime }_{t}\right)}^{-}$ are positive and negative parts of the derivatives, and $v_{P_{1}},\;v_{P_{2}},\;v_{Q},\;v_{A}$ are corresponding velocities of proliferative phase 1, proliferative phase 2, quiescent phase and necrotic phase, respectively. $Q\left(t^{-}\right)$ implies the density of quiescent phase in previous time step. Boundary conditions for turning one phase to another are shown in equation (9).

(9) $$\left\{ \begin{array}{c} P_{1}\left(a=0\right)=2P_{2}\left(a=a_{\max,P_{2}}\right)\\ P_{2}\left(a=0\right)=gP_{1}\left(a=a_{\max,P_{1}}\right)+{\left({g\prime }_{t}\right)}^{+}Q\left(t^{-}\right) \end{array}\right.$$

The cell birth provides a passive movement and activates tumor cell motility. Given the fact that cells move due to passive movement created by the cell birth and are pushed out through the domain boundaries, equation (10) is dedicated to evolution of healthy cells.

(10) $$\frac{\partial M}{\partial t}+\nabla .\left(v_{M}M\right)=0$$

The duplication of cells increases pressure locally and forces cells to move and relax their pressure. The Darcy law for porous media is applied to relate the pressure created by the cell birth and the velocity of tumor cells in tumor microenvironment (equation (11)).

(11) v=−∇φ

in which *v* is velocity of tumor cells and *φ* is a potential corresponding to the pressure in tumor microenvironment. It is assumed that all tumor cells move with the same velocity as $v_{P_{1}}=v_{P_{2}}=v_{Q}=v_{A}=v_{M}=v$ . Combining equations (5)–(8) and (10) provides the relationship shown in equation (12), which is an indicator of tumor expansion.

(12) $$\nabla .v=P_{2}\left(a=a_{\max,P_{2}}\right)$$

### Angiogenesis

Tumor-induced angiogenesis, growth of new capillaries from pre-existing vessels, was almost neglected until the time when Folkman^7^ proposed the idea that angiogenesis is the most fatal part of a tumor’s growth. Many scientists have tried to model angiogenesis based on mathematical models. The one which was proposed by Anderson and Chaplain^42^ has attracted more attention and used by many researchers to simulate angiogenesis. The angiogenesis model in this paper also makes use of formulations represented by Anderson and Chaplain.^42^ Based on Anderson and Chaplain,^42^ there are three mechanisms governing ECs movement in the domain. Random movement which is a consequence of random walk of ECs which has led to a formulation similar to diffusion mechanism. The other mechanism affecting ECs in the extracellular matrix (ECM) which is proved by Keller and Segel^43^ is chemotaxis, interpreted as direct movement of ECs as a result of soluble chemoattractants. The remaining mechanism in this model is haptotaxis, which is representative of transverse movements and is a result of adhesion force in the ECM. Combining these mechanisms and related formulations, total flux term for EC density in the domain is reached as equation (13):

(13) $$J_{n}=J_{\mathrm{random}}+J_{\mathrm{chemo}}+J_{\mathrm{hapto}}$$

In which $J_{n}$ is total flux of ECs, $J_{\mathrm{random}},\;J_{\mathrm{chemo}}$ , and $J_{\mathrm{hapto}}$ are diffusion, chemotaxis, and haptotaxis terms, respectively. Simplifying and writing the related parameters for each term results in equation (14) as below:

(14) $$\frac{\partial n}{\partial t}=D_{n}\nabla^{2}n-\nabla .\left(\chi \left(c\right)n\nabla c\right)-\nabla .\left(\rho_{0}n\nabla f\right)$$

where *n* is density of ECs, *D_n_* is diffusion coefficient of ECs, *χ(c)* is chemotactic function, *c* is VEGF concentration, $\rho_{0}$ is positive coefficient for haptotaxis and *f* is fibronectin concentration. To nondimensionalize equation (14), the related parameters are divided by reference parameters introduced in Anderson and Chaplain^42^ as $\tilde{c}=\frac{c}{c_{0}}$ , $\tilde{f}=\frac{f}{f_{0}}$ , $\tilde{n}=\frac{n}{n_{0}}$ , and $\tilde{t}=\frac{t}{\tau }$ . Using these nondimensional parameters, equation (14) is nondimensionalized and a new dimensionless equation is reached:

(15) $$\frac{\partial n}{\partial t}=D\nabla^{2}n-\nabla .\left(\frac{\chi }{1+\alpha c}n\nabla c\right)-\nabla .\left(\rho n\nabla f\right)$$

Nondimensional parameters in equation (15) are:

(16) $$D=\frac{D_{n}}{D_{c}},\chi =\frac{\chi_{0}c_{0}}{D_{c}},\alpha =\frac{c_{0}}{k_{1}},\rho =\frac{\rho_{0}f_{0}}{D_{c}},\tau =\frac{L^{2}}{D_{c}}$$

Most of the parameters are adopted from Anderson and Chaplain^42^ except the parameters which are related to domain size and should be updated to fit this model. τ is dimensionless time parameter which is dependent upon the distance between the tumor and parent vessel and should be varied based on tumor position in the domain. The related dimensionless time parameters for different distances are stated in the Supplemental File.

Discretizing equation (15) using Euler finite difference scheme, equation (17) is reached:

$$\begin{array}{c} n_{l,m}^{q+1}=n_{l,m}^{q}R_{0}+n_{l+1,m}^{q}R_{1}+n_{l-1,m}^{q}R_{2}+n_{l,m+1}^{q}R_{3}\\ \;+n_{l,m-1}^{q}R_{4} \end{array}$$

$$\begin{array}{c} R_{0}=1-\frac{4\mathrm{kD}}{b^{2}}+\frac{k\alpha \chi \left(c_{l,m}^{q}\right)}{4b^{2}\left(1+\alpha c_{l,m}^{q}\right)}\left[{\left(c_{l+1,m}^{q}-c_{l-1,m}^{q}\right)}^{2}+{\left(c_{l,m+1}^{q}-c_{l,m-1}^{q}\right)}^{2}\right]\\ \;-\frac{k\chi \left(c_{l,m}^{q}\right)}{b^{2}}\left(c_{l+1,m}^{q}+c_{l-1,m}^{q}-4c_{l,m}^{q}+c_{l,m+1}^{q}+c_{l,m-1}^{q}\right)\\ \;-\frac{k\rho }{b^{2}}\left(f_{\;l+1,m}^{\;q}+f_{l-1,m}^{q}-4f_{l,m}^{q}+f_{l,m+1}^{q}+f_{l,m-1}^{q}\right) \end{array}$$

$$\begin{array}{c} R_{1}=\frac{\mathrm{kD}}{b^{2}}-\frac{k}{4b^{2}}\\ \;\left[\chi \left(c_{l,m}^{q}\right)\left(c_{l+1,m}^{q}-c_{l-1,m}^{q}\right)+\rho \left(f_{\;l+1,m}^{\;q}-f_{l-1,m}^{\;q}\right)\right] \end{array}$$

$$\begin{array}{c} R_{2}=\frac{\mathrm{kD}}{b^{2}}+\frac{k}{4b^{2}}\\ {}[\chi \left(c_{l,m}^{q}\right)\left(c_{l+1,m}^{q}-c_{l-1,m}^{q}\right)+\rho (f_{\;l+1,m}^{\;q}-f_{\;l-1,m}^{\;q})] \end{array}$$

$$\begin{array}{c} R_{3}=\frac{\mathrm{kD}}{b^{2}}-\frac{k}{4b^{2}}\\ \left[\chi \left(c_{l,m}^{q}\right)\left(c_{l,m+1}^{q}-c_{l,m-1}^{q}\right)+\rho \left(f_{l,m+1}^{\;q}-f_{l,m-1}^{\;q}\right)\right] \end{array}$$

(17) $$\begin{array}{c} R_{4}=\frac{\mathrm{kD}}{b^{2}}+\frac{k}{4b^{2}}\\ {}[\chi \left(c_{l,m}^{q}\right)\left(c_{l,m+1}^{q}-c_{l,m-1}^{q}\right)+\rho (f_{l,m+1}^{\;q}-f_{l,m-1}^{\;q})] \end{array}$$

An EC’s movement is decided based on this equation in which *R_0_* is the probability to stay stationary and *R*_1_, *R*_2_, *R*_3_, *R*_4_ are probabilities to move right, left, up and down, respectively. *l*, *m* are indicators of position of a point in the discretized computational grid in the direction of *x* and *y*, respectively. These probabilities are normalized by the sum of the probabilities. Then each movement probability falls in a range between 0 and 1. Therefore each limited range represents a movement to one of the four directions and also staying stationary. A random number is generated between 0 and 1 to decide about the direction of movement. Therefore, EC moves in the direction where the random number has fallen.

As stated above, fibronectin exists in the ECM. It adheres to the matrix and does not diffuse in the tissue. Therefore, its concentration is constant in the tissue initially. It is proved that fibronectin is secreted by ECs and also exists in the domain. On the other hand, fibronectin is consumed as ECs move in the domain toward the tumor. Summing all these factors, equation (18) is reached in which there are generation and consumption terms in the formulation:

(18) $$\frac{\partial f}{\partial t}=\omega n-\mu \mathrm{nf}$$

where ω and µ are positive constants.

For ECs to move in the domain, a gradient of VEGF is required. The ECs sense the gradient and move toward the highest gradients. VEGF is consumed while ECs move in the domain which affects their subsequent move. Therefore, the equation of consumption of VEGF is considered in angiogenesis section as below:

(19) $$\frac{\partial c}{\partial t}=-\lambda \mathrm{nc}$$

in which λ is a positive constant. Secretion of VEGF by tumor cells is proposed in the next sections.

Equations (18) and (19) are nondimensionalized using the aforementioned parameters of reference EC density, VEGF, fibronectin, and time constants:

$$\frac{\partial c}{\partial t}=-\eta \mathrm{nc}$$

(20) $$\frac{\partial f}{\partial t}=\beta n-\gamma nf$$

where η, β, and γ are positive non-dimensional coefficients defined as:

(21) $$\eta =\frac{\lambda L^{2}n_{0}}{D_{c}},\;\gamma =\frac{\mu L^{2}n_{0}}{D_{c}},\;\beta =\frac{\omega L^{2}n_{0}}{f_{0}D_{c}}$$

The set of equations (20) is also discretized using Euler finite difference method.

Posterior to cell movement, ECs may encounter each other during movement and fuse together, which will lead to anastomosis. An EC can also come across a stalk cell and anastomosis will occur. On the other hand, ECs may branch in their way toward tumor. Many factors affect EC branching which can be introduced as: (1) ECs and stalk cells must reach a certain age and be mature enough prior to being able to branch, (2) Density of ECs in the point of branching is high enough, and (3) there is an available space nearby for the new EC to occupy. If these conditions are satisfied, one another parameter will be checked to let ECs branch, concentration of TAF must be above a threshold value to let ECs branch.

(22) $$\left\{ \begin{array}{c} P_{\mathrm{branching}}(x,y)=0,\;c(x,y)\leq 0.1\\ P_{\mathrm{branching}}(x,y)=1,\;c(x,y)>0.1 \end{array}\right.$$

The probability condition is estimated in this paper to make the model able to mimic real physical situations in the tumor microenvironment. As stated above, ECs encounter each other and form closed loops. Circulating blood flow is the main aim of tumor-induced angiogenesis to deliver oxygen and nutrients to the tumor site. Therefore, as closed loops form, blood flows in the network and provides fresh oxygen for tumor cells. Dewhirst et al.^44,45^ stated that there is no relationship between vessel diameter and blood flow inside tumor-induced neovessels. Nevertheless, many scientists^39,46–48^ have assumed that since blood flow is laminar inside the neovessels, one can use Poiseuille’s law from Mechanical Engineering formulations to obtain the flow in the vascular network. Therefore, in this research the same procedure is followed and blood is assumed to flow laminarly in the capillaries and obey Poiseuille’s law:

(23) $$\overset{\cdot }{V}=\frac{\pi R^{4}}{8L_{c}}\frac{\Delta P_{B}}{\mu_{B}}$$

where Δ*P_B_* is pressure difference between two junctions, *R* is vessel radius, *L_c_* is distance between two junctions, and µ_*B*_ is blood viscosity. The blood viscosity is assumed constant and equal to 4 × 10^−3^ Pa.s.^49^ Apart from blood flow rate, pressure difference is also unknown in this equation, hence another equation is required to determine the unknowns. Conservation of mass law is applied for each junction of the vascular network where three capillaries meet.

(24) $$\sum_{k=1}^{N}{\overset{\cdot }{V}}_{\left(l,m\right),k}=0$$

where *N* is the number of capillaries joining junction in the position shown by indices (*l*, *m*). Pressure difference across parent vessel is 8000 Pa (60 mmHg) and diameter of parent vessel is 14 µm and new capillaries are 8 µm.^49^

### Vascular growth

**Figure 2.**
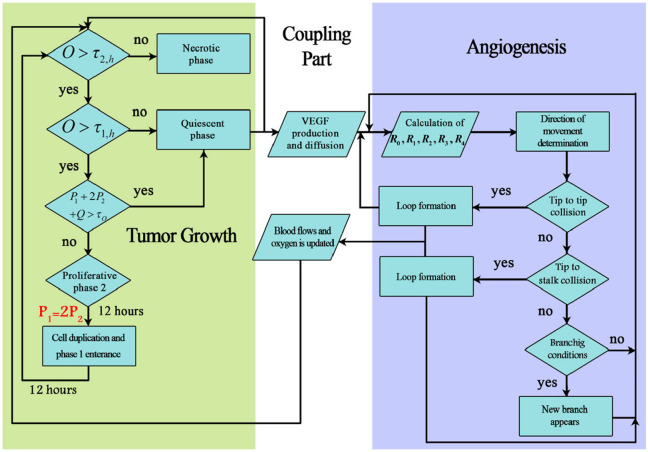
The flowchart for the simulation steps of the coupled tumor growth and angiogenesis. The VEGF and oxygen are used to connect distinct parts of the angiogenesis, tumor growth, and ECM. Oxygen is used to determine the state of tumor cells whereas VEGF provides a gradient of chemoattractant for ECs to move toward the tumor.

Two distinct parts of tumor growth are dependent upon each other via communicating through chemicals in the tumor microenvironment. Among many factors being transmitted via ECM between tumor cells and vessels are nutrients and TAFs. The most dominant TAF here is Vascular Endothelial Growth Factor (VEGF) which is secreted by hypoxic tumor cells and consumed by ECs. On the other hand, vessels are sources of oxygen which is considered to be dominant nutrient in the model. As new capillaries anastomose and blood circulates in the domain, the required oxygen for hypoxic tumor cells are provided and the cells will enter proliferative phase if the conditions are suitable. Flowchart of simulation of vascular tumor growth is demonstrated in Figure 2.

Dissemination of these chemicals are so fast in comparison to tumor growth and angiogenesis phenomena that the equations for concentration of them can be considered to be in steady state conditions. For distribution of VEGF in the domain, equation (25) is proposed as below:

(25) $$0=\frac{\partial c}{\partial t}=\nabla .\left(D_{V}\nabla c\right)+\alpha_{V}Q-\delta_{V}c$$

where *D_V_* is VEGF diffusion rate in the tissue, *α_V_* is production rate of VEGF by quiescent cells, and *δ_V_* is degradation rate of VEGF in the tissue. There is no consumption term in this equation since it is considered in equation (19) where ECs consume VEGF when they move in the domain. The governing equation for calculating oxygen concentration is defined as equation (26).

(26) $$\left\{ \begin{array}{c} 0=\frac{\partial O}{\partial t}=D_{O_{2}}\nabla^{2}O-\left(\beta_{1,O}P+\beta_{2,O}Q+\beta_{3,O}M\right)\\ \;O-\delta_{O}O\\ O=O_{\max},\;\mathrm{where}\;n=1 \end{array}\right.$$

where $D_{O_{2}}$ is diffusion coefficient of oxygen, and *β*_1,*O*_, β_2_*_,O_*, β_3,*O*_ are consumption rates of proliferative, quiescent, and healthy cells, respectively. δ_*O*_ is degradation rate of oxygen within the tissue domain. Required parameters are tabulated in Table 1.

**Table 1.** Parameters involved in equations governing the coupled tumor growth-angiogenesis model.

Parameter	Value(dimension)	References	Explanation	Equations
Tumor growth parameters				
$a_{\max,P_{1}}$	12 h	Billy et al.^33^	Duration of proliferative phase 1	(1), (3), (7)–(9)
$a_{\max,P_{2}}$	12 h	Billy et al.^33^	Duration of proliferative phase 2	(1), (3), (9), (12)
*τ_1,h_*	5.5 M	Höckel and Vaupel^50^	Hypoxia threshold	(1)
*τ_2,h_*	1.52 M	Billy et al.^33^	Severe hypoxia threshold	(2)
*τ_O_*	0.8 cells mm^−2^	Billy et al.^33^	Overpopulation threshold	(1)
*D_V_*	1.044 × 10^−1^ mm^2^ h^−1^	Anderson and Chaplain^42^	VEGF diffusion rate	(25)
α_*V*_	2.11M cells^−1^ mm^2^ h^−1^	Billy et al.^33^	VEGF production rate	(25)
δ_*V*_	1.25×10^−4^ h^−1^	Billy et al.^33^	VEGF degradation rate	(25)
$D_{O_{2}}$	1 mm^2^ h^−1^	Androjna et al.^51^	Oxygen diffusion rate	(26)
*O*_max_	8 M	Pittman^52^	Oxygen concentration in vessels	(26)
β_*1,O*_	3 cells^−1^ mm^2^ h^−1^	Billy et al.^33^	Rate of oxygen consumption by proliferative cells	(26)
β_*2,O*_	1.5 cells^−1^mm^2^ h^−1^	Billy et al.^33^	Rate of oxygen consumptionby hypoxic cells	(26)
β_*3,O*_	3.75 × 10^−1^ cells^−1^ mm^2^ h^−1^	Billy et al.^33^	Rate of oxygen consumptionby healthy cells	(26)
δ_*O*_	10^−3^ h^−1^	Billy et al.^33^	Oxygen degradation rate	(26)
*N_0_*	2 × 10^6^ cells mm^−2^	Ribba et al.^32^	Constant number of cells per unit volume	(4)
Angiogenesis parameters				
*D*	0.00035	Anderson and Chaplain^42^,Lesart et al.^53^	Diffusion coefficient of ECs	(15)
α	1.8	Estimated	Denominator coefficient of chemotactic function	(15)
β	0.45	Anderson and Chaplain^42^,Lesart et al.^53^	Fibronectin production rate	(20)
γ	0.9	Anderson and Chaplain^42^,Lesart et al.^53^	Fibronectin consumption rate	(20)
ρ	0.16	Stéphanou et al.^54^	Haptotaxis coefficient	(15)
χ	1.14	Estimated	Chemotaxis coefficient	(15)
η	0.9	Anderson and Chaplain^42^,Lesart et al.^53^	VEGF consumption rate	(20)

### Numerical method

To explore the effects of domain size (corresponding to tissue size), two different sizes of the domain are selected. The small domain is selected to be 4 mm × 4 mm with 200 × 200 grids and the large domain is selected to be 6 mm × 6 mm with 300 × 300 grids. For ECs with the diameter of 8–12 µm, the average diameter of 10 µm has been selected.

The discrete part of equations that belongs to angiogenesis are discretized using Euler finite difference method. The configuration of domains provides the mesh size of Δ*x* = Δ*y* = *b* = 20 µm. ECs are assumed to move one double space in real tissue during each time step of the simulation to enable us to use the same computational grid for the equations of tumor growth and angiogenesis. Time step is set equal to 1 h when we multiply time step to non-dimensional time parameter. A meshing network of 300 × 300 is used to preserve the size of meshes for the large domain. Therefore, every procedure explained for the small domain is also valid for the large domain.

No-flux boundary condition is imposed on ECs for angiogenesis equation (equation (27)).

(27) $$\zeta .\left(-D_{n}\nabla n+n\left(\chi \left(c\right)\nabla c+\rho_{0}\nabla f\right)\right)=0$$

where ζ is an outward unit normal vector. Finite volume method is used to solve equations of tumor growth. Cell-centered method is used for discretization of equations. The small and large domains consist of 200 × 200 and 300 × 300 control volumes, respectively. Control volumes are chosen such that center points of control volumes coincide with the points used to solve discrete equations of angiogenesis and eliminate the need to interpolation among the points. Since there is no specific restrains for selecting time step of the finite volume method, for the distances of 1, 1.5, and 2 mm time step is equal to 1 h and for the distances of 2.5 and 3 mm, time steps are 2 and 3 h, respectively, when we multiply the nondimensional time step to the nondimensional time parameter. Hence, in order to solve the coupled equations, for each step of tumor cell growth in cases of 2.5 and 3 mm distance, we go through 2 and 3 steps in angiogenesis, respectively.

Boundary conditions for transition among phases are detailed in section 2.1. For the outer boundary, it is assumed that healthy cells leave the domain as tumor grows.

Oxygen and VEGF are used to couple two distinct parts of tumor growth. Steady state equations are used for oxygen and VEGF since their diffusion time scale is much shorter than the time scale of tumor growth and angiogenesis. Equations (25) and (26) are solved using Gauss-Seidel iteration method in the domain. We assume that oxygen and VEGF cannot cross the boundary, therefore Neumann boundary condition is imposed on the edges of the domain for oxygen and VEGF ( $\frac{\partial O}{\partial \zeta }{|}_{\partial \Omega }=0$ and $\frac{\partial c}{\partial \zeta }{|}_{\partial \Omega }=0$ ).

Each configuration has simulated at least for five times and the mean values are reported in the results section.

### Initial conditions

Tumors with a quiescent core and a proliferative rim are considered as initial tumors for simulations. In proliferating rim, outer strip is in proliferative phase 2 and inner strip is in proliferative phase 1. To realize how the size of initial tumor affects angiogenesis process and tumor growth is influenced by angiogenesis, tumor growth and angiogenesis are studied for three initial tumor sizes of small (0.5 mm in diameter), medium (1 mm in diameter) and large (1.5 mm in diameter). Inspired from animal models, different distances between initial tumor and parent vessels (1, 1.5, 2, 2.5, and 3 mm) are examined. Also two different numbers of initial sprouts (three and five sprouts) are tested. Three different conditions are selected for location of vessels with respect to position of initial tumor: (1) only one parent vessel exists on left wall of the domain, (2) two parent vessels exist on left and right walls of the domain, and (3) one parent vessel is placed inside the domain since tumors positioned near the outer boundary may grow beyond the size of domain. To compare the results in different conditions, the distance between initial sprouts is preserved in both of the domains.

It is assumed that fibronectin is initially secreted by ECs and its concentration is higher near vessels and less near the tumor site. Initial profile of fibronectin concentration is defined as equation (28)^42^:

(28) $$f\left(x,y,0\right)=k_{f}e^{-\frac{x^{2}}{\varepsilon_{1}}}$$

where $\varepsilon_{1}$ and *k_f_* are 0.45 and 0.75, respectively. VEGF is distributed within the tissue primarily by quiescent core of the tumor. Since the equations governing angiogenesis are solved in non-dimensional format, values for VEGF and fibronectin concentrations are normalized. Initial VEGF concentration is correlated to initial number of quiescent tumor cells. Initial concentrations of fibronectin and VEGF for different conditions are presented in Supplemental Figures S1 and S2, respectively.

## Results

**Figure 3.**
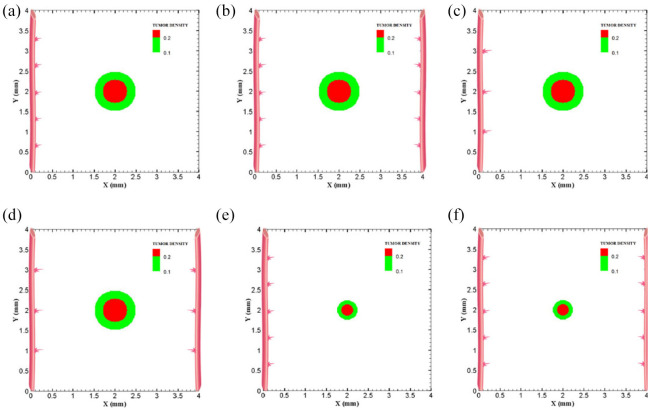
Different configurations of initial conditions in terms of tumor size and number of sprouts in small domain (4 mm by 4 mm): (a) initial medium tumor with one parent vessel and five sprouts, (b) initial medium tumor with two parent vessels and five initial sprouts on each vessel wall, (c) initial medium tumor with one parent vessel and three sprouts on vessel wall, (d) initial medium tumor with two parent vessels and three initial sprouts on each vessel wall, (e) initial small tumor with five initial sprouts on one parent vessel, and (f) initial small tumor with two parent vessels and five initial sprouts on each vessel wall.

**Figure 4.**
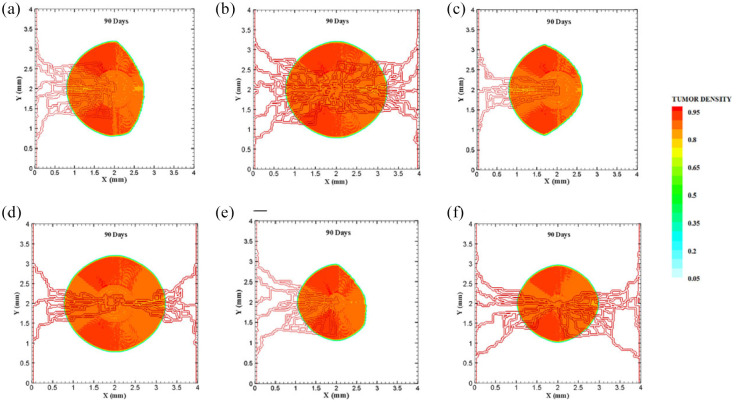
Different configurations of tumors after 90 days of progression with different tumor sizes and number of sprouts in small domain: (a) final configuration of medium size tumor with one parent vessel and five sprouts, (b) final configuration of medium size tumor in the presence of two parent vessels with five initial sprouts on each one, (c) final configuration of medium size tumor with one parent vessel and three sprouts, (d) final configuration of medium size tumor with two parent vessels and three initial sprouts, (e) final configuration of small size tumor with five initial sprouts on one parent vessel, and (f) final configuration of small size tumor with two parent vessels and five initial sprouts.

**Figure 5.**
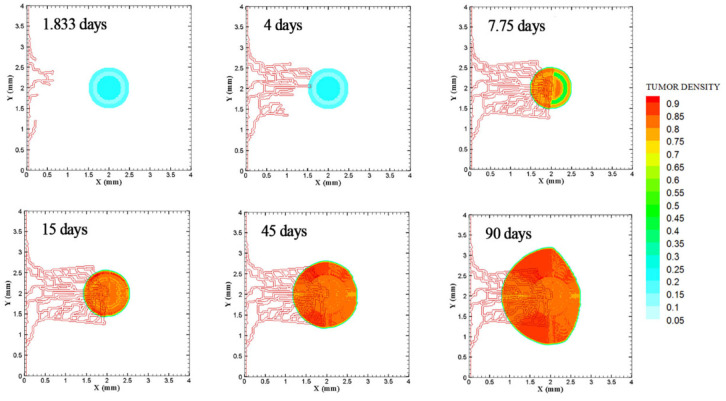
The growth of medium tumor during 90 days in tumor microenvironment with one parent vessel on left wall of 4 mm × 4 mm small domain. First loop adjacent to parent vessel is formed after 1.833 days. Angiogenesis continues for 7.833 days. Tumor continues the growth unconditionally until day 90.

The coupled model of tumor growth and angiogenesis in a vascular tumor microenvironment is established to explore how configuration and properties of parent vessels and initial tumor size and distance regulate tumor growth and angiogenesis. To examine differences among different conditions, the evolution of tumor growth and angiogenesis are presented in 90 days. First, the tumor with following conditions are modeled: initial size of medium (1 mm diameter); the tumor placed at middle of the small domain (4 mm by 4 mm); five sprouts on each parent vessel; and one or two parent vessels placed on walls of the domain (Figure 3(a) and (b)). For the configuration with one single parent vessel on left wall of the domain (Figure 3(a)), branching occurs after 1.3 days and first loop is formed after 1.833 days. The tumor lacks oxygen in first days and all tumor cells turn to hypoxic. Left part of the tumor starts proliferating after 4.5 days and right part of the tumor initiates proliferation after 6.5 days when the capillary network is close enough to the tumor to feed nutrient and oxygen. It should be noted that capillary network reached the tumor in 4 days but capillaries that have reached the tumor site are not functional since they have not anastomosed. Therefore, there is no blood flow in these capillaries. Following propagation of capillary network and formation of closed loops, oxygen diffuses to all parts of tumor and induces proliferation. After a few days of tumor growth when tumor reaches a certain size and consumes oxygen supplied by vessels, farther parts of tumor cannot receive enough oxygen to survive, and therefore they turn to hypoxic cells. The growth of tumor is then biased toward the oxygen source since the right part of the domain does not have access to enough oxygen. Angiogenesis process continues for 7.833 days. The final configuration of tumor growth and angiogenesis after 90 days is shown in Figure 4(a). The results of tumor growth over the course of 90 days for the condition shown in Figure 3(a), with one parent vessel present on left wall and tumor centered in the small domain, are presented in Figure 5 and Supplemental Video 1. The density of tumor cells in initial days of tumor growth increases in the outer rim (where cells are in proliferative phase 2) and cell cycles continue disregarding oxygen availability. As time passes and capillaries provide oxygen for cells on inner parts, tumor also start proliferating until the space is filled without any free space remained for cell duplication. Afterward, cells on a thin strip of proliferating rim on the outer part of the tumor duplicate and move outward, which leads to tumor expansion.

**Figure 6.**
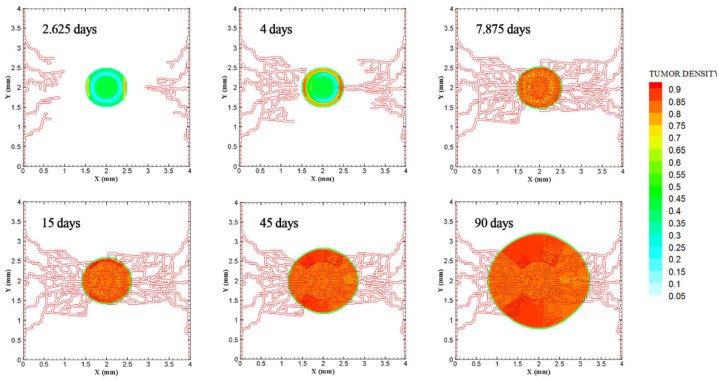
Evolution of medium tumor during 90 days in tumor microenvironment with two parent vessels on left and right walls of 4 mm × 4 mm small domain. Primary capillary loop is formed near right parent vessel in 2.25 days. First loop adjacent to left parent vessel is formed in 2.625 days.

Through incorporation of one more parent vessel to the right wall (Figure 3(b)), oxygen level in right and left rim of tumor reaches above the hypoxic level and a small portion of tumor starts proliferating at first days. Then, the capillary network propagates and reaches tumor and through preparation of fresh oxygen, all tumor cells start proliferating. Branching initiates after 1 day near right parent vessel. First loop is formed after 2.625 days adjacent to right parent vessel. The other parts of tumor that are in hypoxic state start proliferating after 4 days. The capillary network reaches tumor after 4 days and angiogenesis continues for 7.875 days. The results of 90 days of tumor progression and angiogenesis are illustrated in Figure 4(b). The results of tumor growth and angiogenesis over the course of 90 days are presented in Figure 6 and Supplemental Video 2.

Furthermore, to explore how initial number of sprouts affect tumor growth and angiogenesis, initial number of sprouts is reduced from five to three. Although the simulation process remains the same for each tumor, we study differences in timing of events and explain possible deviations from normal tumor growth discussed above. All information about models as well as references to figures and videos are tabulated in Tables 2 and 3. The conditions for tumor and parent vessels with three sprouts are shown in Figure 3(c) and (d). When there is one parent vessel on the left wall (Figure 3(c)), first branch is observed after 1.125 days and first loop is formed after 2.9 days. However, the tumor remains in hypoxic state until it starts proliferating after 4.5 days when the capillary network supplies enough oxygen. The capillary network reaches tumor after 3.625 days and angiogenesis continues for 6.75 days. As tumor grows, the induced capillary network cannot supply oxygen to right parts of tumor and hence the growth is not eccentric and is directed toward parent vessel and capillaries. Capillary network and tumor growth after 90 days are shown in Figure 4(c). The simulation results of tumor growth and angiogenesis for this configuration over the course of 90 days are illustrated in Supplemental Video 3. When there are two parent vessels with three sprouts on each wall (Figure 3(d)), first branch appears after 0.875 days. First loop is formed after 2.5 days. Other parts of the tumor start proliferating after 3.75 days. Capillaries reach the tumor site after 3.75 days but these capillaries are void of blood flow until they form closed loops. Angiogenesis continues for 6.5 days. Tumor growth and capillary network for this condition after 90 days is shown in Figure 4(d). The simulation results of tumor growth and angiogenesis over the course of 90 days are illustrated in Supplemental Video 4. Comparing the configurations with three and five initial sprouts show that capillary density is reduced for three-sprout configuration, therefore we continue the simulations with parent vessels that have five sprouts.

**Table 2.** Characteristics of different configurations of tumor and parent vessels.

Configuration no.	Domain	Tumor	No. of parent vessels	No. of initialsprouts	Distance of vessels and tumor	Initialconfigurationfigure
1	Small	Medium	1	5	2	3-A
2	Small	Medium	2	5	2	3-B
3	Small	Medium	1	3	2	3-C
4	Small	Medium	2	3	2	3-D
5	Small	Small	1	5	2	3-E
6	Small	Small	2	5	2	3-F
7	Small	Small	1	5	1	7-A
8	Small	Small	1	5	1.5	7-B
9	Small	Medium	1	5	1	7-C
10	Small	Medium	1	5	1.5	7-D
11	Small	Small	1	5	2.5	7-E
12	Small	Medium	1	5	2.5	7-F
13	Small	Medium	1	5	3	7-G
14	Large	Medium	1	5	3	9-A
15	Large	Medium	2	5	3	9-B
16	Large	Large	1	5	3	9-C
17	Large	Large	2	5	3	9-D

**Table 3.** Results of different configurations of tumor and parent vessels.

Configuration no.	First branch	First loop	Vascularnetworkand tumorcontact	Angiogenesis duration	Finalconfigurationfigure	Supplemental Video no.
1	1.3	1.833	4	7.833	4-A	1
2	1	2.25	4	7.875	4-B	2
3	1.125	2.9	3.625	6.75	4-C	3
4	0.875	2.5	3.75	6.5	4-D	4
5	2.375	1.875	4.625	8.5	4-E	5
6	2.125	2.125	5	7	4-F	6
7	0.33	1.75	1.875	4.25	8-A	7
8	0.875	2.125	2.875	6.25	8-B	8
9	0.5	1.75	1.042	4.75	8-C	9
10	1.625	3.5	3.125	5.625	8-D	10
11	6.5	11.25	12.25	16.875	8-E	11
12	4.75	10	10.375	15.2	8-F	12
13	5.75	14.25	20.5	26	8-G	13
14	7	10.5	20.25	30	10-A	14
15	9	11	20.5	30	10-B	15
16	6	8	18.125	23	10-C	16
17	6.5	8.5	19	28	10-D	17

To understand how tumor size and initial number of cancer cells affect angiogenesis and tumor growth, the model is simulated for a smaller tumor size with the initial diameter of 0.5 mm, with one and two parent vessels and with five initial sprouts. Initial placement of the tumor and parent vessels are shown in Figure 3(e) and (f). When there is one parent vessel on left wall of the domain (Figure 3(e)), all tumor cells enter quiescent phase. First branching occurs after 2.375 days and first loop is formed after 1.875 days. Left part of the tumor starts proliferating after 5 days while the right part remains hypoxic. Capillary network reaches the tumor site after 4.625 days and right part of the tumor starts proliferating after 6.75 days. Angiogenesis continues for 8.5 days. Similar to one single parent vessel configuration, tumor grows eccentrically toward capillary network. Tumor density and capillaries after 90 days are shown in Figure 4(e) and the simulation results of tumor growth and angiogenesis over the course of 90 days are illustrated in Supplemental Video 5. For the configuration with two parent vessels placed on right and left walls of the tumor domain (Figure 3(f)), right and left rim of the tumor start proliferating from the beginning. First branch is produced adjacent to left parent vessel after 2.125 days and simultaneously first loop forms next to right parent vessel. Since there exist two parent vessels, initial loop is sufficient to feed oxygen to the tumor and induce tumor growth. The capillary network reaches the tumor site after 5 days from both sides. Angiogenesis continues for 7 days (Figure 4(f)). The simulation results of tumor growth and angiogenesis over the course of 90 days in this condition are illustrated in Supplemental Video 6.

**Figure 7.**
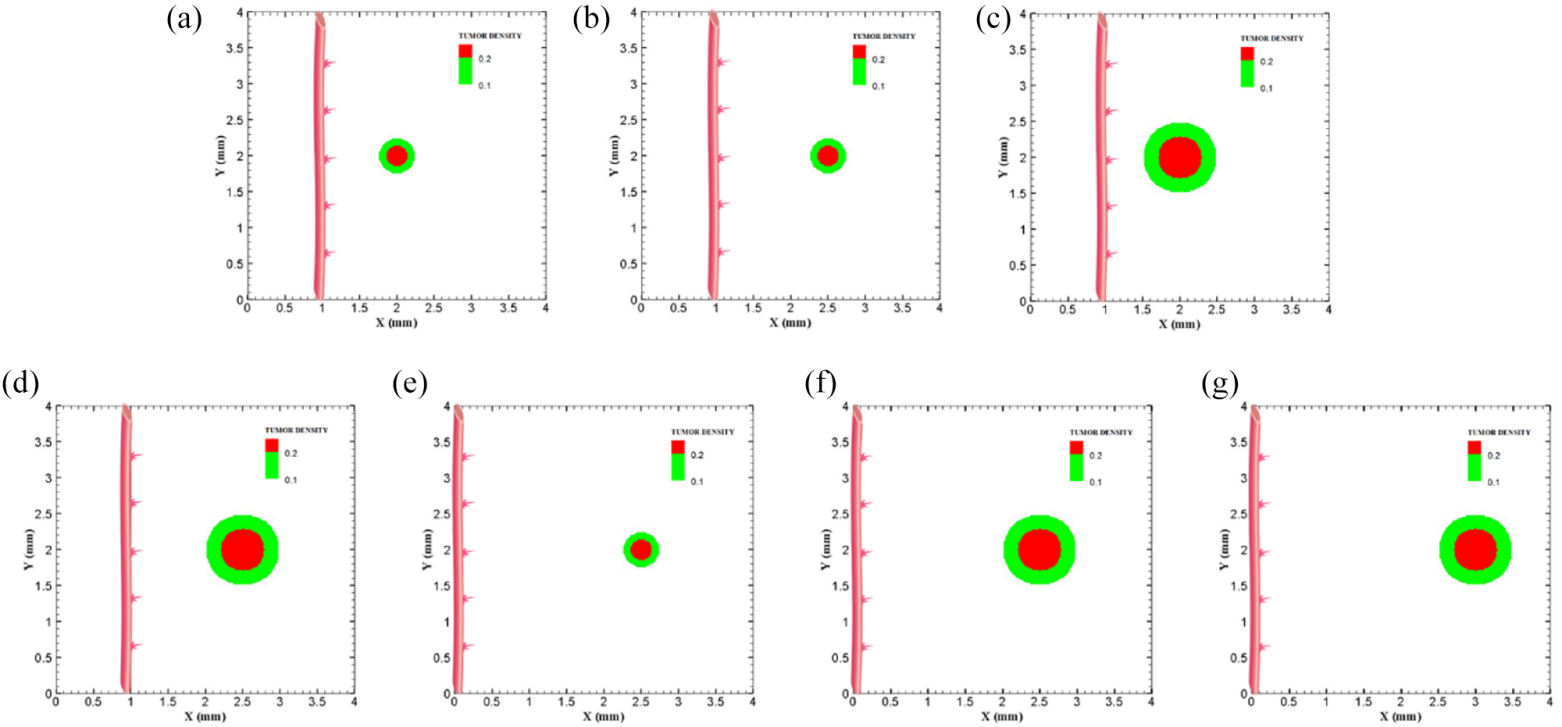
Different configuration of initial conditions in terms of tumor size and position of parent vessels with respect to the tumor with small domain: (a) initial small tumor with one parent vessel 1 mm away, (b) initial small tumor with one parent vessel 1.5 mm away, (c) initial medium tumor with one parent vessel placed 1 mm away, (d) initial medium tumor with one parent vessel with 1.5 mm distance to the tumor, (e) initial small tumor with one parent vessel 2.5 mm away, (f) initial medium tumor with one parent vessel 2.5 mm away, and (g) initial medium tumor with one parent vessel 3 mm away.

**Figure 8.**
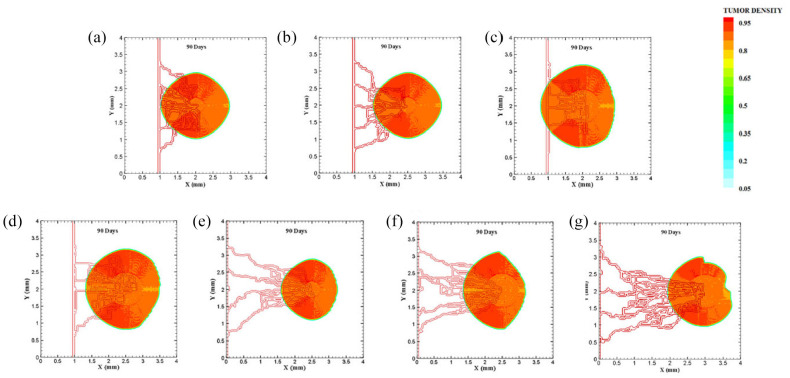
Different configuration of tumors after 90 days of progression in different tumor sizes and position of parent vessels with respect to tumors in small domain: (a) final configuration of small tumor with one parent vessel placed 1 mm away, (b) final configuration of small tumor placed 1.5 mm away from parent vessel, (c) final configuration of medium tumor with one parent vessel 1 mm away, (d) final configuration of medium tumor with one parent vessel 1.5 mm away, (e) final configuration of small tumor with one parent vessel 2.5 mm away, (f) final configuration of medium tumor with one parent vessel 2.5 mm away, and (g) final configuration of medium tumor with one parent vessel 3 mm away.

To investigate how distance between tumor and parent vessel affects tumor growth and angiogenesis, tumors with initial small and medium size are placed closer and farther from the parent vessel which the results are reported in Table 3 for brevity. When tumor with medium size is placed 3 mm away from the parent vessel, the trend of tumor growth is completely different from other configurations (Figure 7(g)). First branches are observed after 5.75 days. First loop is formed after 14.25 days and angiogenesis continues for 26 days. Particularly, right part of the tumor becomes necrotic at first days because of its large distance from oxygen source. Final configurations after 90 days are shown in Figure 8(g), where the results show that tumor growth is directed toward oxygen source in the domain, approaching the newly formed capillary network. Also for the tumor with a distance of 3 mm away from the parent vessel, the duration of angiogenesis increases dramatically and this is due to necrotic region of the tumor with limited production of TAFs (Supplemental Video 13).

**Figure 9.**
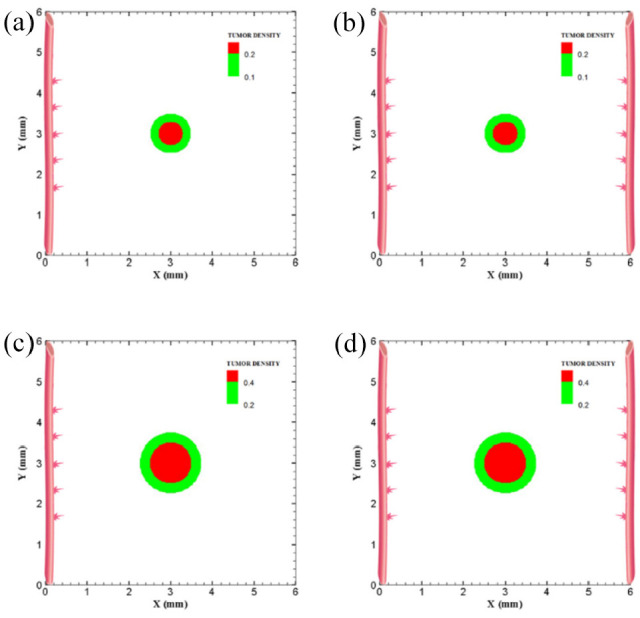
Different configuration of initial conditions in terms of tumor size in large domain: (a) initial medium tumor with five sprouts on one parent vessel, (b) initial medium tumor in the presence of two parent vessels with five initial sprouts on each vessel wall, (c) initial large tumor with five sprouts on one parent vessel, and (d) initial large tumor in the presence of two parent vessels with five initial sprouts on each vessel wall.

**Figure 10.**
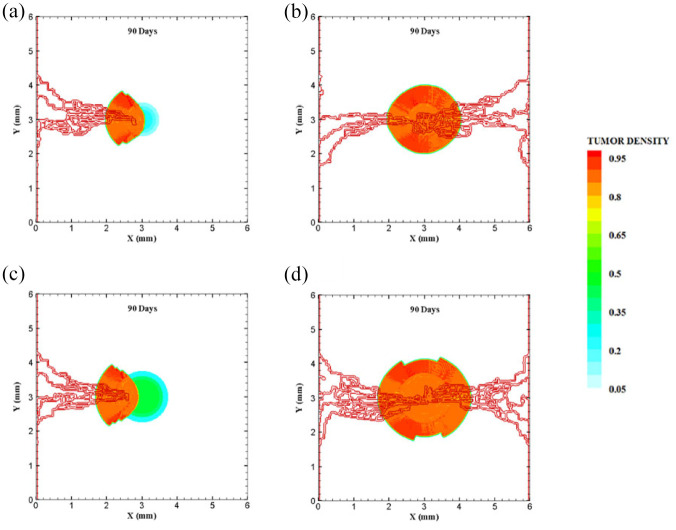
Different configuration of tumors after 90 days of progression with different tumor sizes: (a) final configuration of medium tumor with five sprouts on one parent vessel, (b) final configuration of medium tumor in the presence of two parent vessels with five initial sprouts, (c) final configuration of large tumor with five sprouts on one parent vessel, and (d) final configuration of large tumor in the presence of two parent vessels with five initial sprouts.

The last configuration is to investigate effects of domain size and tumor initial density on vascular tumor growth. First, the centered tumor with an initial medium size and a parent vessel on left wall of the large domain (6 mm × 6 mm) is simulated (Figure 9(a)). First branch is formed 7 days after initiation of angiogenesis and first loop is formed after 10.5 days. Angiogenesis continues for 30 days. The large size of tumor domain reduces tumor growth due to limited oxygen concentration around the capillary network, and therefore make right part of the tumor necrotic (Figure 10(a) and Supplemental Video 14). Incorporation of another parent vessel on right wall (Figure 9(b)) prevents turning tumor cells to necrotic phase. First branching occurs after 9 days and first loop is formed after 11 days. Second loop is produced after 15 days and it takes 30 days for angiogenesis to form final capillary network (Figure 10(b) and Supplemental Video 15). Tumor grows eccentric and all tumor cells receive required oxygen during 90 days of progression.

For the large domain (6 mm by 6 mm), the chemotactic strength reduces as the produced VEGF needs to diffuse a longer distance, therefore gradients of VEGF are weak near parent vessels. Since the chemotactic strength within the large domain is limited, the strategy of increasing the tumor density and as a consequence, the number of tumor cells by five folds is applied to make the tumor larger and realize how tumor growth and angiogenesis are impacted. Tumor with initial large size of 1.5 mm in diameter is placed at center of the large domain (Figure 9(c)). For the configuration with a single parent vessel, first branches are observed after 6 days and first loop is formed after 8 days. Angiogenesis also continues for 23 days. Although we enlarged the tumor and rearranged all sprouts to move toward the tumor, the capillary network cannot supply enough oxygen to cells to induce centric tumor growth and fold around the capillary network. The tumor and capillary network are shown after 90 days in Figure 10(c) and illustrated in Supplemental Video 16. Finally, even by adding another parent vessel on right wall (Figure 9(d)), no necrotic part is observed in the tumor site. Branching initiates after 6.5 days adjacent to right parent vessel and first loop is formed after 8.5 days next to right parent vessel. Capillaries reach tumor after 21 days but angiogenesis continues for 28 days. Sprouts on parent vessels could sense VEGF gradient and move toward the tumor. However, the capillary network could not supply sufficient oxygen to all parts of tumor, therefore a heterogeneous growth of tumor is observed in this configuration (Figure 10(d) and Supplemental Video 17).

**Figure 11.**
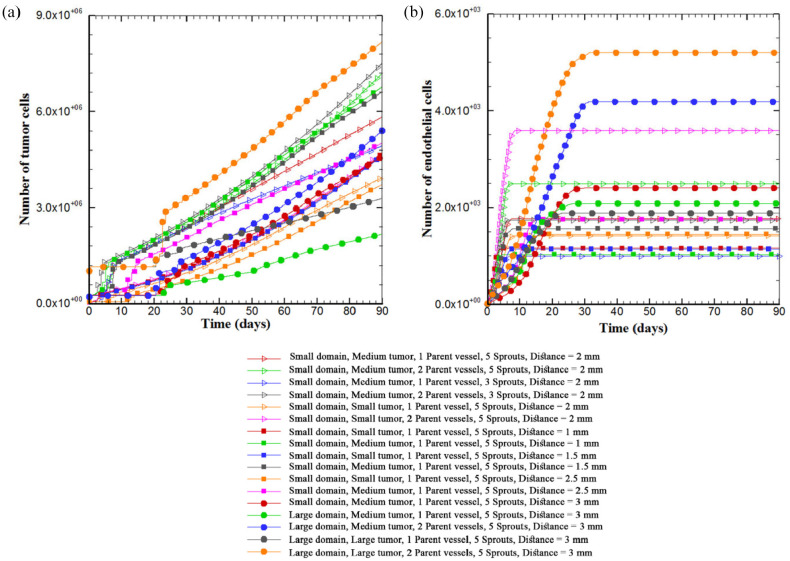
The number of tumor cells and ECs over the course of 90 days of tumor growth: (a) the number of tumor cells versus time over the course of 90 days. Different trends of tumor growth are observed in initial days of simulation for different configurations of tumor microenvironment. Overall, the tumor size increases over time with an increasing curve slope. For the far tumor, the oxygen concentration is not sufficient for proliferation of tumor cells, leading to a hypoxic condition. This implies a discontinuity in tumor proliferation for some days. Also one part of the tumor away from blood source becomes necrotic and (b) the number of ECs in the capillary network for 90 days. The number of ECs at first days of growth increases slowly but the rate increases during vascular branching toward the tumor. The rate of increase in the number of ECs then slows down due to a decrease in branching and anastomosis. Lastly, angiogenesis stops completely and the number of ECs remains unchanged.

The number of tumor cells is plotted versus time to compare the trends of tumor growth for different architectures of parent vessels, initial tumor and size of domain (Figure 11(a)). The results confirm that the pattern of tumor growth, when it is fed sufficiently by vessels, obeys the power law of tumor growth, reported by others^55,56^ and proved in Ghazani et al.^41^ A variety of different growth patterns is observed depending upon their conditions. The diversity of patterns of tumor growth is mainly resulted from gradients of oxygen in microenvironment which dictates biased growth of tumor toward oxygen source and drop of proliferating rim of the tumor. Rapid initial growth rate in first days is due to sufficient space available for cells to grow inside the tumor. When the cells continue proliferation, they are packed inside tumor which increases the density of tumor and enforces the movement toward proliferating rim. Thereby, the rate of tumor growth becomes proportional to the rate of growth in proliferating rim. The larger the tumor diameter, the higher the rate of tumor growth, demonstrated by the increase in slope of growth curve over time (Figure 11(a)). For quiescent state of the tumor, growth is paused for several days but reinitiates once it is fed by new capillaries. This discontinuity is more obvious in asymmetric conditions of the far tumor in the presence of only one parent vessel. In this condition, a necrotic part is formed in the tumor site and the number of tumor cells stays unchanged for several days. However, the growth is recovered after receiving enough oxygen. Moreover, for the tumor in large domain in the presence of one parent vessel, the slope of growth curve is less than other conditions given the existence of necrotic part and limited oxygen source for tumor cells.

The number of ECs in the domain for 90 days for different configurations of tumor-vessel is shown in Figure 11(b). The trend of variations in number of ECs is identical for all configurations. The rate of increase in number of ECs is slow in first days but increases during sprouting and branching. When the capillary network reaches the tumor, the rate of increase in number of ECs reduces until angiogenesis stops. There is no change in number of ECs after entry of branches into the tumor. Number of ECs is highest in the large domain with two parent vessels. Density of ECs in small tumor configuration is higher than large tumor because ECs have more space near the tumor to branch and anastomose.

## Prediction of tumor cells number by artificial neural network

Artificial neural networks have a structure similar to the human brain, which have been widely used in cancer therapy applications.^57,58^ The brain, as an information processing system, is made up of key structural elements called neurons. Artificial neural networks also contain a set of interconnected neurons, called layers.^59^ To create these layers, these neurons are connected by activation (stimulus) functions. In practice, a limited number of functions are used as activation functions, and neural network researchers often prefer to use nonlinear stimulus functions such as Gaussian or tangent hyperbolic as activation functions.^60^ Neural networks, despite their diversity, have a similar structure. A neural network consists of three layers of input, hidden and output layers. The input layer only receives information and acts as an independent variable. Therefore, the number of input neurons depends on the nature of the problem and depends on the number of independent variables. The output layer also acts as a dependent variable, and the number of neurons depends on the number of dependent variables.

**Figure 12.**
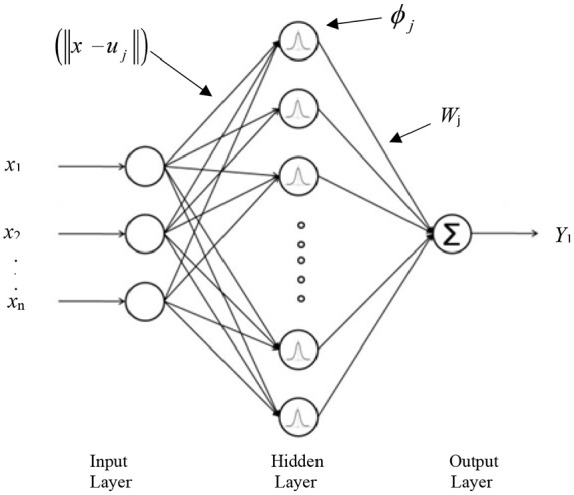
Structure of radial basis function.

Neural networks with radial basis function are widely used to nonparametric estimation of multidimensional functions through a limited set of educational information. Radial neural networks with fast and comprehensive training, are interesting and efficient networks which have received special attention.^61–63^ Hartman et al.^64^ proved that the networks with radial basis function are very powerful approximators. It requires sufficient number of neurons in the hidden layer to make it able to approximate any continuous function with certain accuracy. The interesting aspect is that these networks have this property only with one hidden layer. Radial-based networks derive the most inspiration from the statistical techniques of pattern classification, and their major advantage is the classification of patterns that have nonlinear space. The main RBF architecture consists of a two-layer network such as Figure 12.

The hidden layer establishes a nonlinear adaptation between the inputs and plays an important role in converting nonlinear patterns into linear discriminant patterns. The output layer produces the weighted sum of the linearized patterns along with a linear output. If the RBF is used to approximate the function, such output would be useful; but if patterns need to be classified, then a sigmoid function can be applied to the output nerves to produce output values of 0 or 1. As can be seen from the description above, the unique feature of this network is the process that is performed in the hidden layer. The output of the RBF network can be calculated by equation (29).

(29) $$F\left(x\right)=\sum_{j=1}^{p}w_{j}\phi \left(\|x-u_{j}\|\right)$$

Where $w_{j}$ is the weights of each neuron and $u_{j}$ are the centers of the function of each neuron. The famous function in radial networks is a Gaussian or exponential function as follows:

(30) $$\phi \left(\|x-u_{j}\|\right)=e^{-{\left(\frac{\left\|x-u_{j}\right\|}{\sigma_{j}}\right)}^{2}}$$

**Figure 13.**
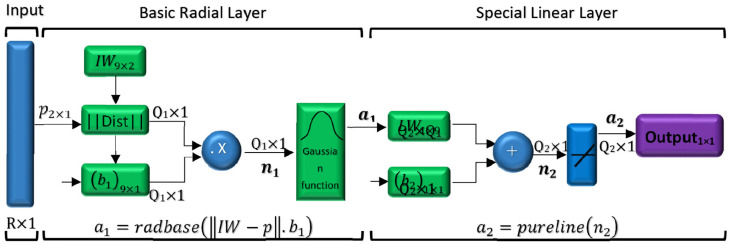
Schematic figure of RBF network designed for estimation number of tumor cells.

In this equation $\sigma_{j}$ is the spread value which is extracted experimentally. The initial size of tumor and distance between tumor and parent vessel are as the inputs of the network and the number of tumor cells is as the output of network. The initial size of tumor, and distance between tumor and parent vessel, calculated in Section 3, have been used as training inputs in the RBF. The schematic of the proposed neural network to predict the tumor volume is represented in the Figure 13.

In this figure *R* is the number of elements in the input vector, *p*_2_ × 1 is the input vector which is made of two elements including initial size of tumor and distance between tumor and parent vessel. Also *Q*_1_ shows the number of neurons in the hidden layer, *Q*_2_ represents the number of neurons in the output layer, *a_1_* demonstrates the output of basic radial layer, and *a*_2_ demonstrates the output of RBF networks. Additionally, *n*_1_ is determined by dot product between weights and bias vectors in hidden layer, and *n*_2_ is the summation of weights vectors and bias in output layer. As it is shown in Figure 13, there are nine neurons in the hidden layer and one neuron in output layer. (IW) and (*b*_1_) are the values of neuron weights and bias in hidden layer, respectively. Also, (LW) and (*b*_2_) are the values of neuron weights and bias in the output layer which are listed in Table 4.

**Table 4.** Parameters of RBF network used for prediction of tumor cells number.

Numbers	*N*_1_	*N*_2_	*N*_3_	*N*_4_	*N*_5_	*N*_6_	*N*_7_	*N*_8_	*N*_9_
Input values	[S, 1]	[S, 1.5]	[S, 2]	[S, 2.5]	[M, 1]	[M, 1.5]	[M, 2]	[M, 2.5]	[M, 3]
Target value	4.65E + 6	4.56E + 6	3.95E + 6	3.73E + 6	6.80E + 6	6.65E + 6	5.85E + 6	5.00E + 6	4.56E + 6
IW	[1, 1]	[1, 1.5]	[1, 2]	[1, 2.5]	[2, 1]	[2, 1.5]	[2, 2]	[2, 2.5]	[2, 3]
LW	−6.42E + 6	11.3E + 6	−11.1E+6	3.23E + 6	3.15E + 6	−2.16E + 6	2.58E + 6	0	−1.17
*b*_1_	0.832	0.832	0.832	0.832	0.832	0.832	0.832	0.832	0.832
*b*_2_	5.12E + 6								

By substituting the above values in equation (31), for every input value (*p*), the network is able to predict the output values as follows:

(31) $$\mathrm{output}=\left({\left[\mathrm{LW}\right]}_{1\times n}\times {\left[e^{-\left(\sqrt{\sum_{k=1}^{i}{\left(IW_{j}\left(k\right)-p\left(k\right)\right)}^{2}}•{\left[b_{1}\right]}_{n\times 1}\right){\;}^{2}}\right]}_{n\times 1}\right)+b_{2}$$

In this equation, *j* represents the *j*-th row of weight matrix in the hidden layer, *k* shows the *k*-th element of weight vector and the input vector, *i* is the number of elements in the weight vector, and *n* is the number of neurons in layer 1. Also, in this network the value of spread is considered to be σ = 1. By considering the above equation and substituting any desired value for tumor distance and initial tumor size in the specified range of acceptable inputs as described in Table 4, the number of tumor cells can be estimated after 90 days of growth. Hence, in order to evaluate the accuracy of the predicted values by equation (31), four arbitrary inputs are given to the network as test inputs and the results of the network are compared to the corresponding results of the mathematical model. The error of the RBF network is demonstrated in Table 5.

**Table 5.** Error percentage between exact and prediction values.

	Small tumor withdistance 1.75	Small tumorwith distance 2.25	Medium tumorwith distance 1.25	Medium tumorwith distance 2.75
Exact value	4.18E + 06	3.88E + 06	5.79E + 06	4.42 + E06
Prediction value	4.39E + 06	4.1E + 06	6.03E + 06	4.73E + 06
Error percentage	5	5.6	4.1	7

**Figure 14.**
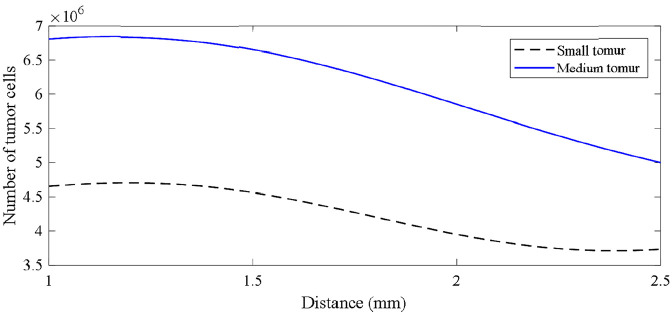
Number of tumor cells evolution vs distance for small and medium initial tumors.

The results indicate the high accuracy of the network which is 95%. Using the proposed neural network, the number of tumor cells are predicted at different distances of tumor and the parent vessel in Figure 14. The distance is varied between 1 and 2.5 and the curves for both small and medium tumors are depicted.

According to these results, increasing the distance between tumor and parent vessel leads to a decrease in the number of tumor cells. Also, the rate of decrement for the number of tumor cells in short distances between (1 and 1.5 mm) is less than longer distances (1.5–2.5 mm).

## Discussion and conclusions

A new multiscale age-structured cell cycle model coupled with a discrete agent-based angiogenesis method is proposed. Four different phases of tumor cells were incorporated to model cell cycle, including two proliferative phases, quiescent phase and necrotic phase. Different tumor-parent vessel arrangements were simulated in configurations where initial tumor has grown to such a certain size that existing vasculature is unable to provide required oxygen. The scenario demonstrates the situation when tumor induces angiogenesis with the focus on dynamic features of tumor cells and capillary network, population, and density of tumor cells, and sprouting and angiogenesis. In addition to mathematical modeling, an artificial neural network is adopted to be trained for available results to propose an overall formulation for the mathematical model. This formulation would be useful in determining the final number of tumor cells in the domain.

As stated in Young,^65^ genetic mutations are not the only reason behind tumor cells growth. Many environmental conditions affect tumor growth and can strongly influence the growth of a tumor in oncoming days. With the knowledge of the trend and response of a tumor in subsequent days of growth, it would be possible to suppress and hinder tumor growth. Anti-angiogenesis therapeutics have attracted much attention in recent years. Angiogenesis as the pivotal part of growth of a solid tumor is being studied whether experimentally or numerically. Clinicians are seeking a way to hinder angiogenesis in order to reduce nutrients of the tumor and suppress the growth and metastasis. Prediction of behavior of a tumor in subsequent days in different positions in relation to the tumor will help clinicians better manipulate the therapeutic implementations.

In this study, effects of density of a tumor prior to angiogenesis, number of initial sprouts, and distance of the tumor and parent vessels are investigated. Moreover, to understand the effects of size of the tissue where tumor appears and also in vitro assay cultures, two different domain sizes are studied.

It is demonstrated that the smaller the initial size of the tumor, the stronger the gradient of angiogenic factor is needed to produce sprouts directing straight toward the tumor. The rate of tumor growth for small tumor is less than the rate for large tumor, probably due to a weaker strength of angiogenic chemoattractant gradients. It is also shown that the longer the initial distance of tumor cells to parent vessels, the higher the density of initial tumor is needed to induce sprouting at distant points of vessels.

Even for the configuration of one single parent vessel next to small tumor, if the number of sprouts is adequate and capillary network forms properly, part of the tumor that is not faced to the vessel may receive enough nutrients and oxygen to grow in proliferation state unconditionally. Also the smaller the distance of the tumor to the vessel, the more centric the growth of the tumor.

Approved by many animal models and studied by Folkman,^40^ it is stated that when a tumor placed in distances far away from a parent vessel, for example, 2.5 and 3 mm away, it may take much longer time for the vascular network to fully grow and reach the tumor. In this study, it is also concluded that when a tumor is moved in the distances of 2.5 and 3 mm away from a parent vessel, not only a bigger tumor is required to induce sprouting and subsequent angiogenesis phenomenon but also it will take much longer time in comparison to closer distances to tumor to form a vascular network of blood vessels including blood flow.

The RBF neural network provides a comprehensive formulation to be used in prediction of final tumor status in the domain. Based on this formula, the curves for the final number of tumor cells as a response of a tumor in the domain are plotted against tumor-parent vessel distance. As the results demonstrate, beyond a certain distance, both small and medium tumors weaken and the intensity of tumor cell number decrement increases. This gives us the sense that a tumor is strongly dependent upon vascular network such that in farther distances where vasculature growth is delayed, tumor becomes smaller.

All in all, as it is stated in Perfahl et al.^37^ and Grogan et al.,^38^ the geometry of domain in which tumor grows is pivotal and this new hybrid model enables us to monitor growth of a tumor and predict final architecture of the network in different circumstances. Anti-angiogenesis therapeutics are proved to be effective modalities in preventing devastating growth of a tumor and our model again pinpoints the momentous role of angiogenesis in fatal growth of a tumor. Future studies can enrich our model by introducing dynamic tumor network, lymphocytes, stromal cells, and other nutrients such as glucose into the model. Moreover, since blood vessels in tumor-induced neovasculature is tortuous and badly vascularized, smeared representation of neovasculature can be a tool to model patient-specific angiogenesis models which can be combined with the present model to achieve better outcomes for clinical purposes in the future studies.

## Supplemental Material

sj-pdf-1-pih-10.1177_09544119211028380 – Supplemental material for Mathematical simulation and prediction of tumor volume using RBF artificial neural network at different circumstances in the tumor microenvironment Click here for additional data file. Supplemental material, sj-pdf-1-pih-10.1177_09544119211028380 for Mathematical simulation and prediction of tumor volume using RBF artificial neural network at different circumstances in the tumor microenvironment by Mehran Akbarpour Ghazani, Mohsen Saghafian, Peyman Jalali and Madjid Soltani in Proceedings of the Institution of Mechanical Engineers, Part H: Journal of Engineering in Medicine
