# Supplementary material for: Mathematical simulation and prediction of tumor volume using RBF artificial neural network at different circumstances in the tumor microenvironment
Source: Proc Inst Mech Eng H. 2021 Jul 10;235(11):1335–55. doi: 10.1177/09544119211028380 (PMC8573697; doi:10.1177/09544119211028380)
Supplement: sj-pdf-1-pih-10.1177_09544119211028380 – Supplemental material for Mathematical simulation and prediction of tumor volume using RBF artificial neural network at different circumstances in the tumor microenvironment [file sj-pdf-1-pih-10.1177_09544119211028380.pdf]

Mathematical simulation and prediction of tumor volume using RBF artificial neural network at different circumstances in the tumor microenvironment

Mehran Akbarpour Ghazani[1, 2 *], Mohsen Saghafian[1], Peyman Jalali[2], Madjid Soltani[3, 4, 5, 6 *]

[1] *Department of Mechanical Engineering, Isfahan University of Technology, Isfahan, Iran*

[2] *Faculty of Mechanical Engineering, University of Tabriz, Tabriz, Iran*

[3] *Department of Mechanical Engineering, K.N. Toosi University of Technology, Tehran 1969764499, Iran*

[4] *Advanced Bioengineering Initiative Center, Computational Medicine Center, K. N. Toosi University of Technology, Tehran, Iran*

[5] *Centre for Biotechnology and Bioengineering (CBB), University of Waterloo, Waterloo, Ontario, Canada*

[6] *Department of Electrical and Computer Engineering, University of Waterloo, Waterloo, ON, Canada*

*Corresponding Authors:
M. Soltani, University of Waterloo, 200 University Ave. Waterloo, Ontario, Canada, N2L3G1, Tel. /Fax: +1 (519) 888-4567, Email address: msoltani@uwaterloo.ca
M. Akbarpour Ghazani, University of Tabriz, Email address: mehran.akbarpour@tabrizu.ac.ir

Nondimensional parameters:

An important parameter determining the movement of capillaries towards tumor is chemotactic coefficient, χ_0. Since this coefficient controls the strength of sprout's direct movement toward tumor, it is important to deal with it in different conditions. When tumor and parent vessel are close to each other, strong chemotactic gradient is needed to make the capillaries move toward tumor. As the concentration of VEGF has increased near the tumor and chemotactic nondimensional parameter depends on it, when we compare VEGF concentration near parent vessel for the case of tumor near parent vessel (when tumor is 1 and 1.5 mm away from parent vessel) with other cases, we see that VEGF concentration is three times higher in relation to

other cases. So we triple chemotactic nondimensional coefficient in this case to make sprouts move toward tumor.

Branching Probability:

As it is mentioned in the paper, it is assumed that capillaries can branch if the concentration of VEGF is above a certain value that lets vessels branch in the domain. When we move the tumor closer or farther to the parent vessel, concentration of VEGF becomes higher or lower in the vicinity of parent vessel. Therefore, we have to increase or decrease the concentration limit to make the capillaries branch closer or away from parent vessel. This threshold values are chosen by trial and error to mimic real tumors. In addition to the attempts to simulate the real situations, one interesting result achieved was that this threshold strongly affects the movement of ECs toward the tumor. This is because without branching and making loops, ECs just wander around and cannot stabilize in their way toward the tumor. Therefore, branching and anastomosis have the important role of stabilizing wandering ECs and direct and guide vessels toward VEGF gradient. As a result, this is a crucial point in determining angiogenesis and it is obtained based on a myriad of simulations. The branching distance where makes Endothelial Cells stable and potent to move towards tumor is obtained as follows:

Distance = 1 mm

$$\begin{cases} P_{\text{branching}}(x,y) = 0, & c(x,y) \le 0.3 \\ P_{\text{branching}}(x,y) = 1, & c(x,y) > 0.3 \end{cases} \tag{9}$$

Distance = 1.5 mm

$$\begin{cases} P_{\text{branching}}(x,y) = 0, & c(x,y) \le 0.2 \\ P_{\text{branching}}(x,y) = 1, & c(x,y) > 0.2 \end{cases} \tag{10}$$

Distance = 2.5 mm, 3 mm

$$
\begin{cases}
P_{\text{branching}}(x,y) = 0, & c(x,y) \le 0.1 \\
P_{\text{branching}}(x,y) = 0.5, & 0.1 < c(x,y) \le 0.15 \\
P_{\text{branching}}(x,y) = 1, & c(x,y) > 0.15
\end{cases}
\tag{11}
$$

Initial conditions:

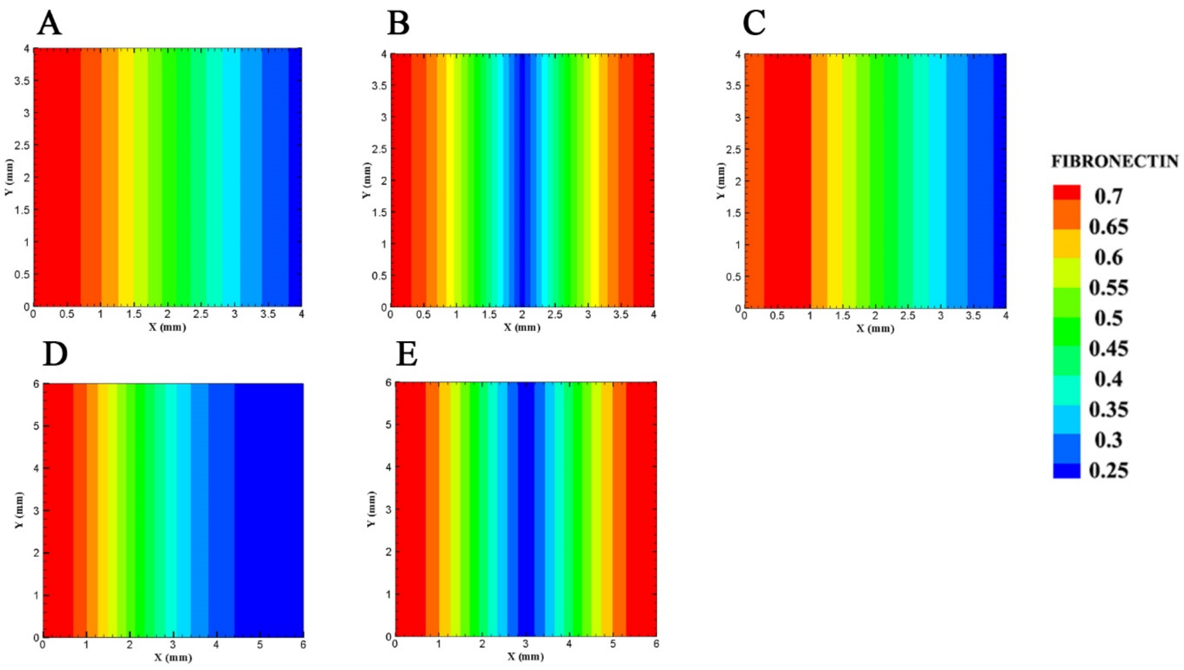

S1 fig. The initial concentration of fibronectin in different conditions. Initial fibronectin concentration in the small domain (**A**) in presence of one parent vessel, (**B**) two parent vessels, and **(C)** in the case of moved vessel inside the domain. Initial fibronectin concentration in the large domain (**D**) in presence of one parent vessel, and (**E**) two parent vessels.

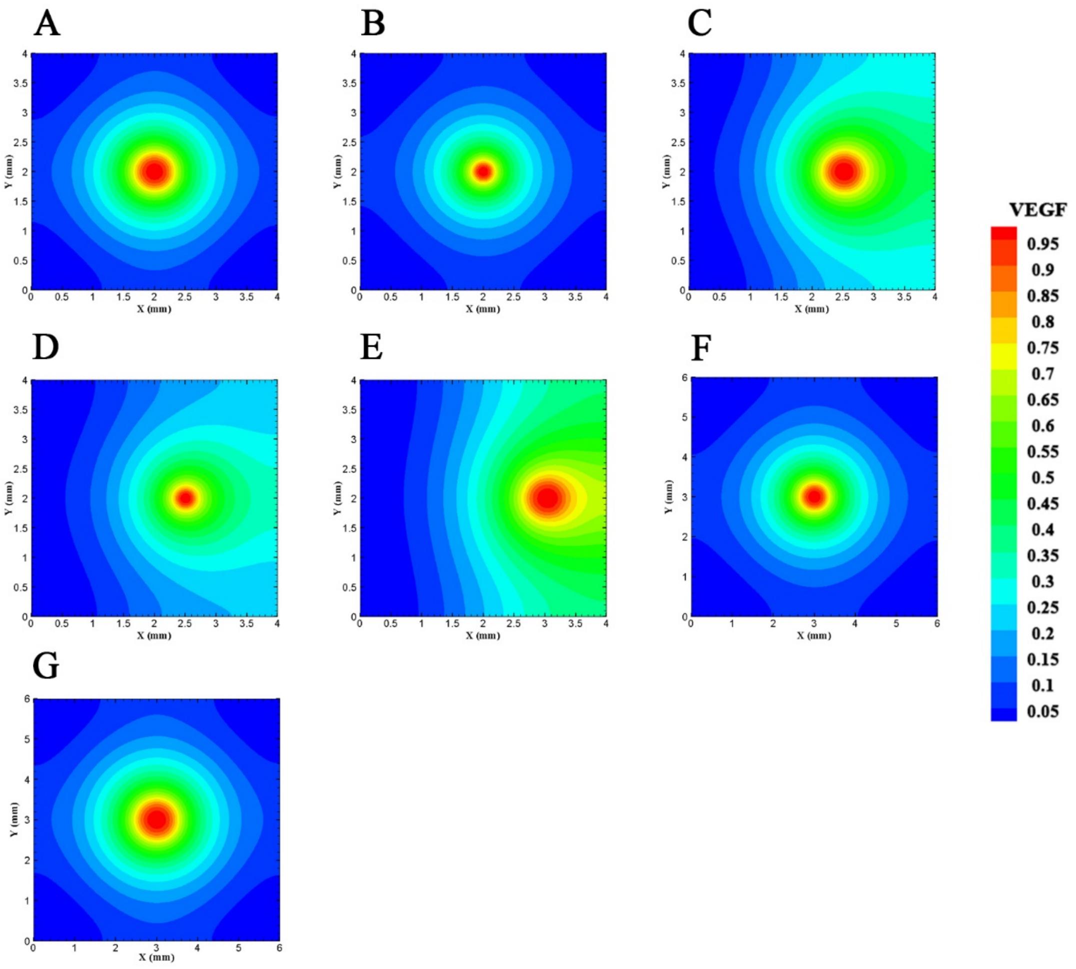

S2 fig. Initial concentration of VEGF in different conditions. (**A**) The initial VEGF concentration secreted by a medium tumor centered in the small domain, (**B**) The VEGF concentration secreted by a small tumor centered in the small domain, (**C**) The VEGF concentration of a medium tumor placed 2.5 mm away from the left wall in the small domain, (**D**) The VEGF concentration of a small tumor placed 2.5 mm away from the left wall in the small domain, **(E)** The VEGF concentration of a medium tumor placed 3 mm away from the left wall in the small domain, **(F)** The secreted VEGF concentration by the medium tumor centered in the large domain, **(G)** The secreted VEGF concentration by the large tumor centered in the large domain.
